# Metal Ion-Directed Specific DNA Structures and Their Functions

**DOI:** 10.3390/life12050686

**Published:** 2022-05-05

**Authors:** Toshihiro Ihara, Yusuke Kitamura, Yousuke Katsuda

**Affiliations:** Division of Materials Science and Chemistry, Faculty of Advanced Science and Technology, Kumamoto University, 2-39-1, Kurokami, Chuo-ku, Kumamoto 860-8555, Japan; ykita@kumamoto-u.ac.jp (Y.K.); katsuda2243@kumamoto-u.ac.jp (Y.K.)

**Keywords:** DNA conjugate, metal ion, triple helix, silver ion, lanthanide, ATP sensor, aptamer, terpyridine, sequence edition, DNAzyme

## Abstract

Various DNA structures, including specific metal ion complexes, have been designed based on the knowledge of canonical base pairing as well as general coordination chemistry. The role of metal ions in these studies is quite broad and diverse. Metal ions can be targets themselves in analytical applications, essential building blocks of certain DNA structures that one wishes to construct, or they can be responsible for signal generation, such as luminescence or redox. Using DNA conjugates with metal chelators, one can more freely design DNA complexes with diverse structures and functions by following the simple HSAB rule. In this short review, the authors summarize a part of their DNA chemistries involving specific metal ion coordination. It consists of three topics: (1) significant stabilization of DNA triple helix by silver ion; (2) metal ion-directed dynamic sequence edition through global conformational change by intramolecular complexation; and (3) reconstruction of luminescent lanthanide complexes on DNA and their analytical applications.

## 1. Introduction

Almost 20 years after the completion of the Human Genome Project [1,2], nucleic acid chemistry is once again a research focus, with the emergence of a number of new fields, including epigenetics, RNA interference, noncoding RNA, mRNA vaccines, iPS cells, and nucleic acid medicine. Molecular engineering in nucleic acid chemistry has become more flexible than ever before to meet the demands and new challenges in these emerging research fields, taking advantage of functional nucleic acids such as aptamers [3], ribozyme [4], and DNAzyme [5,6,7], as well as programmed spontaneous strand exchange reactions such as DNA circuits [8,9].

The basis of any molecular engineering of nucleic acids, after all, is the knowledge and techniques for the formation of canonical and some noncanonical structures of nucleic acids. Under certain conditions, we are now able to logically design a variety of static and dynamic structures of DNA/RNA by predicting the most stable duplex structures that will form in the solutions containing these mixtures. The pioneering work in predicting the thermodynamic stability of duplex structures based on the nearest-neighbor model was undoubtedly revolutionary [10,11,12,13]. These advances have contributed to the development of almost all modern hybridization-based techniques widely used for gene expression control, gene editing, and analysis, such as antisense, RNAi, CRISPR/Cas9, and in situ hybridization, among others.

In our previous work, using synthetic DNAs and DNA conjugates, we reported various conjugates consisting of oligo DNAs and functional molecules, e.g., anthracene [14], β-cyclodextrin [15], ferrocene [16], and several metal ion chelators [17]. The DNA conjugates were programmed or designed to change their structures in various ways in response to specific stimuli. Outputs, including photochemical ligations, luminescence, and electrochemical responses resulting from the structural changes, have been used to detect the stimuli themselves, complementary DNA/RNA, or other biomolecules. The merit of nucleic acids as molecular platforms is that pre-designed structures can be precisely constructed in a bottom-up fashion, providing an unparalleled advantage with respect to all biotechnological applications as mentioned above. This short article summarizes some of the works we conducted after one of the authors (T.I.) visited the laboratory of Prof. Breslauer at Rutgers University in 2001–2002, especially for the systems related to complexation with metal ions. In these works, metal ions were used as a critical structural factor and a signal generator in the design of both static and dynamic DNA structures.

## 2. Stabilization of a Parallel-Motif DNA Triplex by Silver Ion

When designing the ligands for specific sequences in DNA duplexes, triple helix formation is a useful recognition motif, inasmuch as the formation of the base triplet follows the simple rule of complementary Hoogsteen hydrogen bonding, CG.C^+^ and TA.T, for the parallel motif of the triplex. However, the triplexes containing CG.C^+^ triplets form only in a weak acidic solution, because the N_3_ position of cytosines (p*K*_a_ = 4.5) in the third strand must be protonated to fulfill its complementarity [18]. With the aim of achieving sufficient stability under physiological conditions, a large quantity of chemically modified DNA has been developed by taking advantage of the highly advanced techniques of organic synthesis [19].

**Figure 1:**
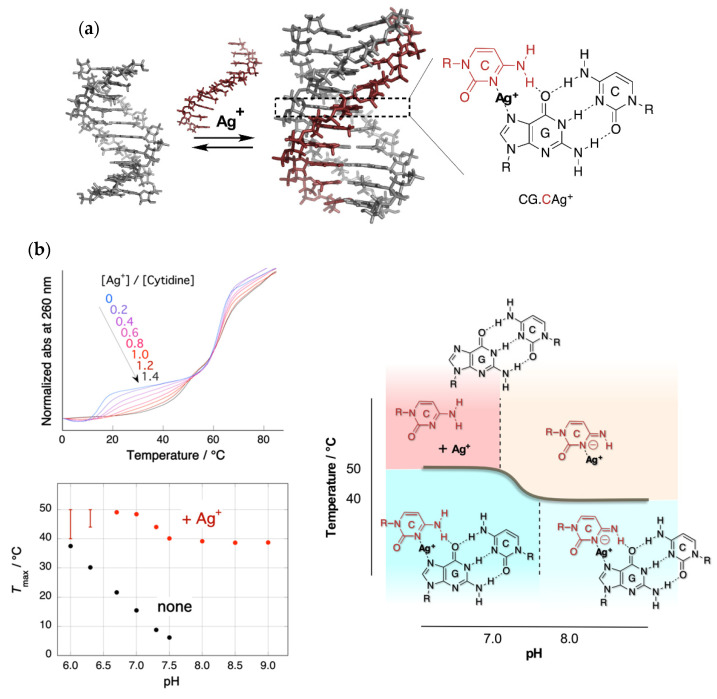
Triplex stabilization by silver ion. (**a**) Structure of the triplex and CG.CAg^+^ base triplet; (**b**) upper left: UV melting curves in the presence of Ag^+^ with various feeding ratios. Only the temperature of the triplex–duplex transition increased with the addition of Ag^+^. Bottom left: pH dependence of the melting temperatures of triplex in the absence and presence of Ag^+^. The melting temperature in the presence of Ag^+^ consists of two pH-independent regions. Right: Phase diagram of the structure of Ag^+^-mediated nucleobase complexes.

We reported an effective alternative method for the stabilization of the parallel motif triple helix of DNA using silver ions (Ag^+^) [20]. Ono et al. reported that the formation of C–C and T–T mispairings in the duplex is promoted by Ag^+^ and Hg^2+^, respectively [21]. In these duplexes, the ions were placed between the bases to form specific bridges (C–Ag^+^–C, T–Hg^2+^–T). These results led to the idea that it might be possible to stabilize triplex structures containing CG.C^+^ base triplexes with Ag^+^. The silver ion was expected to displace an N_3_ proton of a cytosine in the CG.C^+^ to form a metal ion-mediated base triplet, CG.CAg^+^, as shown in Figure 1a. This process was expected to stabilize parallel motif triplexes even at neutral pH.

Figure 1b shows the UV melting curves at a pH of 7.0 and pH dependence of the temperatures of triplex–duplex transition. Surprisingly, the addition of an equal amount of Ag^+^ (to CG.C^+^) increased the melting temperature of the triplex by more than 30 °C under neutral conditions [20]. In the absence of Ag^+^, the relation of the melting temperature to pH was clearly evident. Meanwhile, in the presence of Ag^+^, the correlation disappeared, and a biphasic feature consisting of two temperature-independent regions was observed. A phase diagram of the structure of the Ag^+^-mediated nucleobase complex could be drawn based on this characteristic melting temperature–pH property. Mass spectrometry (ESI-TOF MS) clearly showed the quantitative formation of the Ag^+^-mediated base triplet, CG.CAg^+^. The results of modeling studies by DFT (B3LYP/6-31G*//3-21G) suggest that the cytosines on the third strand are forced to be twisted from the plane of Watson–Crick GC pairs in CG.CAg^+^ triplets, because the coordination distance in N–Ag^+^–N would be longer than that of the Hoogsteen hydrogen bonds, N–H^+^–N, in CG.C^+^. The deviation from the typical triplex structure observed in studies using CD is consistent with this non-planarity of CG.CAg^+^.

The method described here for the stabilization of DNA triplexes is both simple and effective. All that is required is the addition of an equimolar amount of Ag^+^ into the solution containing the DNA triplex. The triplexes mediated by Ag^+^ were found to be stable even in a weak basic solution and can be applied in various research tasks, including the regulation of DNAzyme activity [22], sensing [23,24], and luminous Ag nanocluster formation [25].

## 3. Metal Ion-Directed Dynamic Splicing of DNA through Global Conformational Change by Intramolecular Complexation

**Figure 2:**
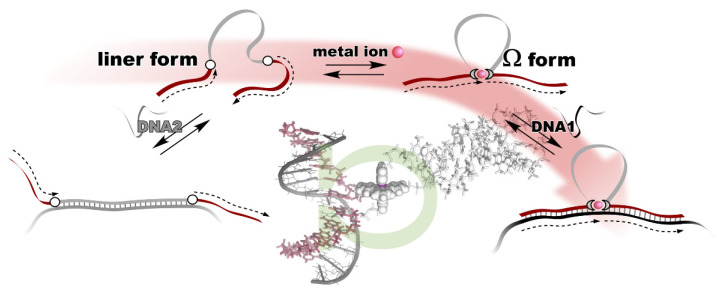
Metal ion-directed reversible edition of the DNA sequence. The sequence of **terpy_2_DNA** is edited by intramolecular complexation with appropriate metal ions through Ω-shaped global conformational change.

The metal ion-directed global conformational control of DNA was performed as follows. Two terpyridine units were built into the distal sites on the DNA backbone to prepare a conjugate, i.e., **terpy_2_DNA**. The two terpyridines formed a stable intramolecular 1:2 complex, [M(terpy)_2_]^2+^, with divalent transition metal ions, M^2+^, namely Fe^2+^, Ni^2+^, Cu^2+^, and Zn^2+^. By the specific formation of an intramolecular metal complex, a part of the sequence of the DNA in between the two terpyridine units was reversibly excluded, and the two flanking external DNA segments were directly connected with each other to form an Ω-shaped structure presenting a new sequence (Figure 2). This can be regarded as a metal ion-directed reversible edition of the DNA sequence or dynamic DNA splicing [26].

Conformational control of **terpy_2_DNA** was confirmed via UV melting with the complementary tandem sequence of the two external segments. The results show that the duplex structure was significantly stabilized in the presence of an equimolar amount (to **terpy_2_DNA**) of transition metal ions. In addition, in the presence of the metal ions, the shape of the melting curves changed to be more cooperative, indicating that the two sequences outside the terpyridines were cooperatively dissociated in a narrow temperature range. The dependences of duplex stabilization on the metal ion feeding ratio (*r* = [M^2+^]/[**terpy_2_DNA**]) were different for each of the metal ions. In the case of Fe^2+^ and Ni^2+^, the duplex remained stable even when additional metal ions were added to *r* = 2 or 3. In contrast, the duplex was destabilized at higher feeding ratios of Cu^2+^ and Zn^2+^. The stability of the duplex was maintained even in the presence of the excess amounts of Fe^2+^ and Ni^2+^, because the Ω-shaped conformation of **terpy_2_DNA** was preserved due to the magnitudes of the two successive binding constants with terpyridine, *K*_1_ < *K*_2_. As for Cu^2+^ and Zn^2+^, the global conformation of **terpy_2_DNA** was changed from Ω-form to a linear form accompanying the transition of the complex types formed on **terpy_2_DNA** from [M(terpy)_2_]^2+^ (on **terpy_2_DNA**∙M^2+^) to 2[M(terpy)]^2+^ (on **terpy_2_DNA**∙2M^2+^) with increasing amounts of ions due to their binding properties with terpyridine, *K*_1_ > *K*_2_. This indicates that the general trend of the complexation of transition metal ions found in the text books of coordination chemistry is still valid on DNA.

**Figure 3:**
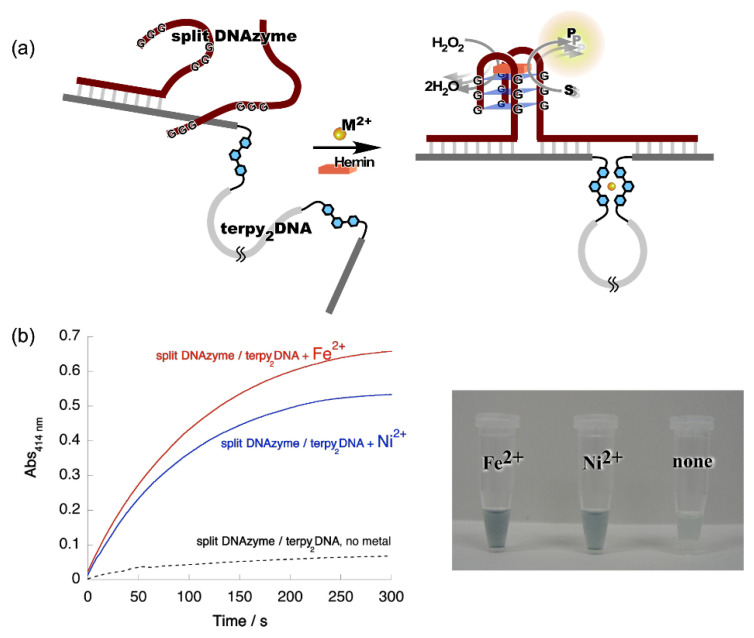
Metal ion-directed regulation of DNAzyme activity. (**a**) Allosteric regulation of split DNAzyme activity by metal ion-directed dynamic sequence edition of the template, **terpy_2_DNA**. (**b**) Left: Time courses of the ABTS oxidation by the split DNAzyme with **terpy_2_DNA** in the presence of Fe^2+^ and Ni^2+^. Red, split DNAzyme/**terpy_2_DNA** + Fe^2+^; blue, split DNAzyme/**terpy_2_DNA** + Ni^2+^; black, split DNAzyme/**terpy_2_DNA**, no metal ions. Right: Images of reaction solutions shown in the time courses.

We then applied the metal ion-directed sequence edition based on the Ω-motif to regulate the function of the split DNAzyme with peroxidase-like activity. To activate the split DNAzyme, they need to be reconstituted to form a G-quadruplex structure. As shown in Figure 3a, **terpy_2_DNA** was used as the tunable template to activate the split DNAzyme. The reaction was monitored by the color change associated with the oxidation of the substrate, 2,2’-azino-bis(3-ethylbenzothiazoline-6-sulfonic acid (ABTS). Figure 3b shows the time course of the reaction profiles. Equivalent concentrations of Fe^2+^ and Ni^2+^ (*r* = 1) restored the activity of the split DNAzyme in the presence of **terpy_2_DNA** [26]. Cu^2+^ and Zn^2+^ also showed a moderate effect on the restoration of split DNAzyme activity. As we expected, the global conformation of **terpy_2_DNA** was fixed to a Ω-shape by the intramolecular formation of [M(terpy)_2_]^2+^. Subsequently, the new sequence presented on **terpy_2_DNA** ∙M^2+^ worked as an effective template to reconstruct the integrated active form of DNAzyme.

The results demonstrated that the global DNA structure and, furthermore, the activity of DNAzyme were controlled by local metal complexation events that could be rationally designed based on general coordination chemistry. The technique of dynamic DNA splicing proposed in this study would be a compatible technique with the construction of the molecular systems consisting of functional DNA, such as aptamer and DNAzyme. Based on the Ω-motif, one could control the activity of reconstituted functional DNA or RNA, thermodynamics and kinetics of strand exchange, and gene expression.

## 4. Reconstruction of Luminescent Lanthanide Complexes on DNA and Their Analytical Applications

**Figure 4:**
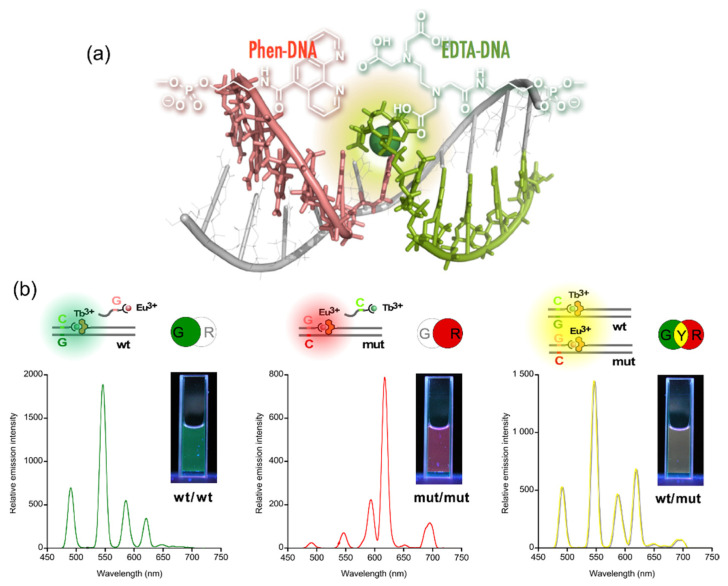
Multicolored allele typing using time-resolved luminescence from lanthanide complexes (Tb^3+^ and Eu^3+^) cooperatively formed with a pair of split probes. (**a**) The structure of the Ln^3+^ complex formed on tandem duplex of the split probes with target sequence. (**b**) Allele typing of thiopurine S-methyltransferase gene.

The present study demonstrated a straightforward genetic analysis using DNA-templated cooperative complexation between a luminescent lanthanide ion (Ln^3+^: Tb^3+^ or Eu^3+^) and two DNA conjugates. Ethylenediaminetetraacetic acid (EDTA) and 1,10-phenanthrorine (phen) were covalently attached to the end of oligo DNAs to form a pair of the conjugates, i.e., capture and sensitizer probes, respectively. The sequences of these split probes were designed so as to form a tandem duplex with targets (templates) with their auxiliary units facing each other, providing a microenvironment to accommodate Ln^3+^ (Figure 4a) [27]. The results of time-resolved luminescence studies showed that the formation of luminous ternary complexes, EDTA/Ln^3+^/phen, depends on the sequence of the targets. The intensity of the luminescence is affected by the binding affinities of the probes or the local structural disruption caused by one-base mispairing [28].

This technique was applied to the multicolored allele typing based on single nucleotide polymorphisms (SNPs) in thiopurine S-methyltransferase gene by the concomitant use of the two capture probes, which are complementary to a part of the wild-type (**wt**) and the mutant (**mut**) of the gene. First, the capture probes for **wt** and **mut** were mixed with equimolar amounts of Tb^3+^ and Eu^3+^, respectively. Both the allele-specific capture probe with Ln^3+^ and the sensitizer probe were then added to three different solutions containing the targets, **wt**/**wt**, **mut**/**mut**, and **wt**/**mut**. The solutions emitted distinctive colors, i.e., green, red, and yellow for **wt**/**wt**, **mut**/**mut**, and **wt**/**mut**, respectively; the colors were identifiable with the naked eye (Figure 4b) [29].

**Figure 5:**
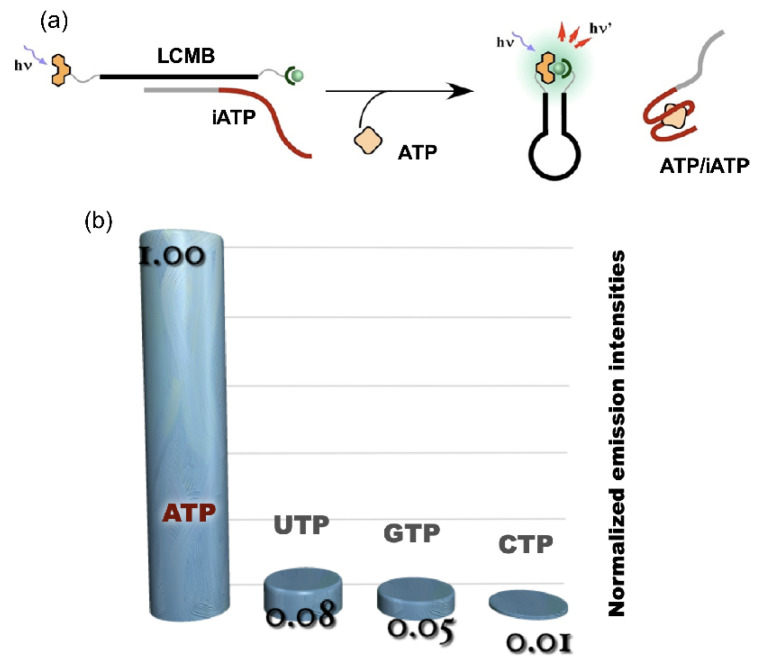
ATP sensing using **LCMB** and **iATP**. (**a**) operating principle of ATP sensing using competitive reaction over **iATP** between ATP and **LCMB**; (**b**) luminescence signal response of ATP sensor to NTPs.

The system was applied as a molecular nanodevice consisting of the lanthanide complex and stem-loop structured oligo DNA. The nanodevice was synthesized by the introduction of EDTA and phen at the 5’- and the 3’-end of the DNA, respectively. This device was named the lanthanide complex molecular beacon (**LCMB**). In the stem-loop structure of **LCMB**, the two auxiliary units were placed in close proximity, providing a microenvironment to accommodate Ln^3+^. The characteristic emissions of Tb^3+^ and Eu^3+^ were clearly observed in the solution containing the nanodevice and the corresponding Ln^3+^ (“on” state). In contrast, scarce emission was observed in the presence of the DNA complementary to the loop region; the auxiliary units were separated from each other when the duplex was formed (“off” state). The ATP aptamer (**iATP**) was used as an interface for the application of **LCMB** to ATP sensing. The sequence of **LCMB** was designed to be complementary to a part of **iATP** (Figure 5a). With the addition of ATP to the **LCMB**/**iATP** duplex, the fluorescence signal turned on as the result of the restoration of **LCMB** stem-loop structure accompanying the displacement of **iATP** from **LCMB** by ATP. A highly specific response was observed for ATP among NTPs, as shown in Figure 5b [30].

**Figure 6:**
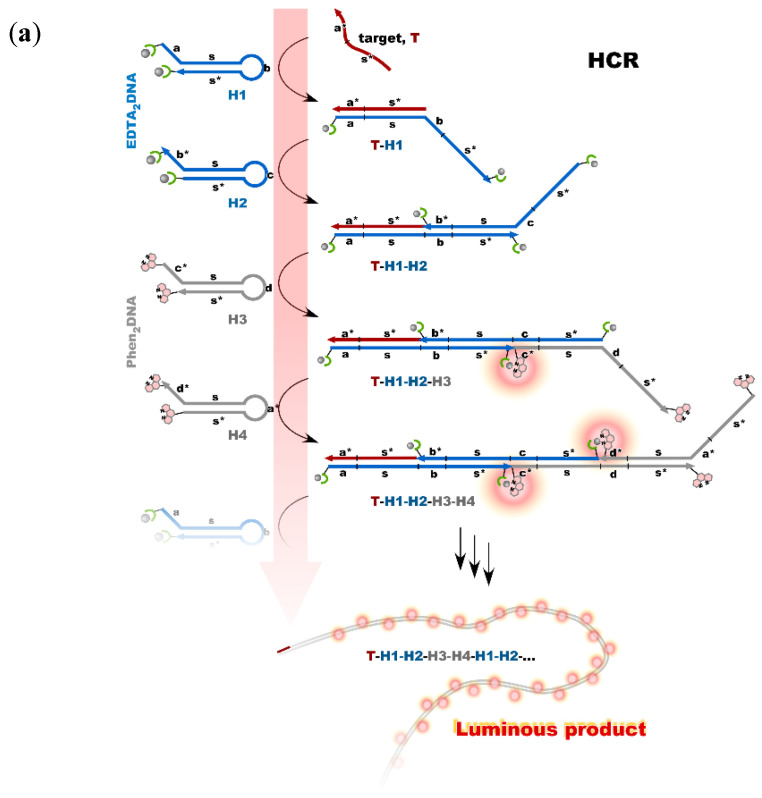

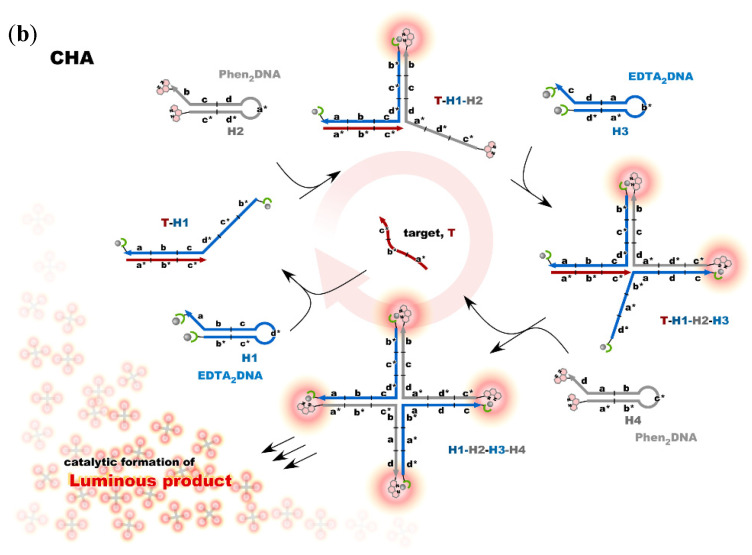
Nonenzymatic signal amplification by DNA circuits: (**a**) Luminous DNA wire was produced by target-initiated HCR; (**b**) Luminous cruciform DNA was produced by CHA.

Nonenzymatic amplification of the luminescent signal from the Ln complexes on the DNA scaffold was performed through catalytic hairpin assembly (CHA) and hybridization chain reaction (HCR), which are the typical DNA circuits consisting of the autonomous successive strand exchange reactions [31,32]. For HCR, four hairpin DNA conjugates were prepared; two of them carry EDTA on both ends, and phens are attached to both ends of another two hairpin strands DNAs. The sequences of the four hairpin DNA strands were designed so as to provide the long DNA wire as the product with Ln complexes at every junction. The HCR was initiated by a small amount of target DNA, acting as an initiator. Figure 6a shows the scheme of the HCR amplification. The luminescence signal significantly increased with the progress of HCR after target addition. Signal contrast was very high, and the sequence selectivity was preserved in this system [32]. To improve the amplification rate, the system was redesigned to form a cruciform product consisting of four hairpins by catalytic hairpin assembly (CHA) (Figure 6b). The sequences of hairpin monomers were modified so as to hybridize convergently to form a closed cruciform structure. Ln complexes were expected to form at each of the four tips of the cruciform. The target miRNA *let-7a* was detected using time-resolved luminescence measurement techniques [32]. The CHA system (cruciform formation) was found to be more efficient than that of the earlier version of HCR (DNA wire), probably due to the difference in molecular sizes of the products.

## 5. Perspective

In recent years, research on nucleic acids has uncovered new and challenging issues as mentioned above, and nucleic acid conjugates show promise as a molecular tool that can be used to meet those challenges. In addition to the standard complementary nucleic acids, functional nucleic acids, such as aptamers and DNAzyme, as well as nonnatural nucleic acids have been added to the options as nucleic acid components of the conjugates. Furthermore, given the diversity of functional molecules that pair with DNAs, an infinite number of combinations are possible in the design of nucleic acid conjugates. With the emergence of “click chemistry”, the in situ synthesis of conjugate molecules is now possible, further expanding the potential of these molecules [33,34]. As demonstrated in the above-referenced studies, it is always critical to accurately predict the structure of nucleic acid conjugates. The fundamentals in the physical chemistry of nucleic acids that Professor Breslauer and his research groups have achieved are pioneering and of universal value. The sum of these works can be considered a milestone in the history of nucleic acid science. We would like to conclude this brief note by sending our best wishes from Japan to Professor Breslauer, on the occasion of his 75th birthday.
